# Detection and mapping of mtDNA SNPs in Atlantic salmon using high throughput DNA sequencing

**DOI:** 10.1186/1471-2164-12-179

**Published:** 2011-04-07

**Authors:** Olafur Fridjonsson, Kristinn Olafsson, Scott Tompsett, Snaedis Bjornsdottir, Sonia Consuegra, David Knox, Carlos Garcia de Leaniz, Steinunn Magnusdottir, Gudbjorg Olafsdottir, Eric Verspoor, Sigridur Hjorleifsdottir

**Affiliations:** 1 Matís, Vínlandsleið 12, 113 Reykjavík, Iceland; 2 Institute of Biological Sciences, University of Wales, Aberystwyth, UK; 3 Department of Pure & Applied Ecology, Swansea University, Swansea SA2 8PP, UK; 4 Freshwater Laboratory, Marine Scotland, Pitlochry, Scotland PH16 5LB, UK

## Abstract

**Background:**

Approximately half of the mitochondrial genome inherent within 546 individual Atlantic salmon (*Salmo salar*) derived from across the species' North Atlantic range, was selectively amplified with a novel combination of standard PCR and pyro-sequencing in a single run using 454 Titanium FLX technology (Roche, 454 Life Sciences). A unique combination of barcoded primers and a partitioned sequencing plate was employed to designate each sequence read to its original sample. The sequence reads were aligned according to the *S. salar *mitochondrial reference sequence (NC_001960.1), with the objective of identifying single nucleotide polymorphisms (SNPs). They were validated if they met with the following three stringent criteria: (i) sequence reads were produced from both DNA strands; (ii) SNPs were confirmed in a minimum of 90% of replicate sequence reads; and (iii) SNPs occurred in more than one individual.

**Results:**

Pyrosequencing generated a total of 179,826,884 bp of data, and 10,765 of the total 10,920 *S. salar *sequences (98.6%) were assigned back to their original samples. The approach taken resulted in a total of 216 SNPs and 2 indels, which were validated and mapped onto the *S. salar *mitochondrial genome, including 107 SNPs and one indel not previously reported. An average of 27.3 sequence reads with a standard deviation of 11.7 supported each SNP per individual.

**Conclusion:**

The study generated a mitochondrial SNP panel from a large sample group across a broad geographical area, reducing the potential for ascertainment bias, which has hampered previous studies. The SNPs identified here validate those identified in previous studies, and also contribute additional potentially informative loci for the future study of phylogeography and evolution in the Atlantic salmon. The overall success experienced with this novel application of HT sequencing of targeted regions suggests that the same approach could be successfully applied for SNP mining in other species.

## Background

Single nucleotide polymorphisms (SNPs), representing single base differences between individuals, are a common form of genome variation [1]. Once identified, SNPs have the potential to be used as genotyping markers for population assignment or in phylogeographic analysis, and are rapidly becoming the marker of choice within this field of study [2]. The emergence of high-throughput (HT) sequencing technologies provides an unparalleled opportunity for the cost-effective sequencing of targeted genomic regions for SNP identification. HT sequencing has been applied for SNP discovery in humans [3,4], animals [5], plants [6,7] and bacteria [8] - in species where reference genomes exist. In organisms lacking a sequenced reference genome, SNPs have also been mined from the random sequencing of either expressed sequence tags (ESTs) [9,10] or reduced representation libraries [11-13]. However, with an available reference sequence, specific genetic regions of interest can be amplified using PCR prior to HT sequencing. The sequence reads of about 400 bp obtained using the Titanium GS FLX chemistry (Roche, 454 Life Sciences) make the 454 sequencing platform particularly suitable for sequencing PCR generated amplicons. This approach involves the emulsion based amplification of individual PCR products and simultaneous, parallel pyrosequencing of DNA strands [14].

Mitochondrial DNA (mtDNA) has been widely used in studies of phylogenetics, molecular ecology and phylogeography in a range of organisms, including the Atlantic salmon based on RFLP or sequences from using Sanger technology [15-17]. As SNPs represent the main form of polymorphism observed in the mtDNA, recent research has been focused upon the identification of a geographically informative mitochondrial SNP panel suitable for high-throughput genotyping (e.g. mtSNP mini-sequencing) [18].

Traditional Sanger-based sequencing methods are relatively expensive and time-consuming, restricting the number of individuals and gene regions that can be sequenced, given the funding available for most studies. Consequently, often a relatively few individuals from restricted geographical areas have been sequenced in order to obtain SNP panels. The SNPs have been subsequently used to screen larger samples from a broader geographical range [1], which has led to low power in resolving lineages and their relationships. It has also raised potentially serious concerns associated with ascertainment bias, such as the situation whereby inferences are significantly conditioned by the gene region screened or the populations used to identify the polymorphisms. The latter could potentially lead to a biased interpretation of the extent to which individuals and populations are related.

HT sequencing technologies offer the potential to overcome these limitations by allowing the rapid and economic sequencing of large genome regions. However, most applications of HT technology to-date have been designed to sequence a large number of fragments from relatively few individuals. In this study, a novel approach was developed by combining the traditional PCR amplification of known gene regions with 454 Titanium FLX (Roche, 454 Life Sciences) sequencing. This approach allowed the sequencing of extensive regions of the mtDNA genome within a broad sample group both quickly and in accordance with a limited budget. The method was tested on the Atlantic salmon (*Salmo salar*) - a fish extensively surveyed using both nuclear [19] and mitochondrial DNA markers, including a mtDNA SNP variation [19-22], and for which 152 nuclear SNPs and 125 mtDNA SNPs [20,22,23] have already been reported. Here, 546 samples from 48 locations throughout the species' geographical range were screened for 7215 bp, encompassing approximately 43% of the mtDNA genome in order to minimize the degree of ascertainment bias relative to previous comparable studies.

## Results

### PCR Amplification

**Figure 1:**
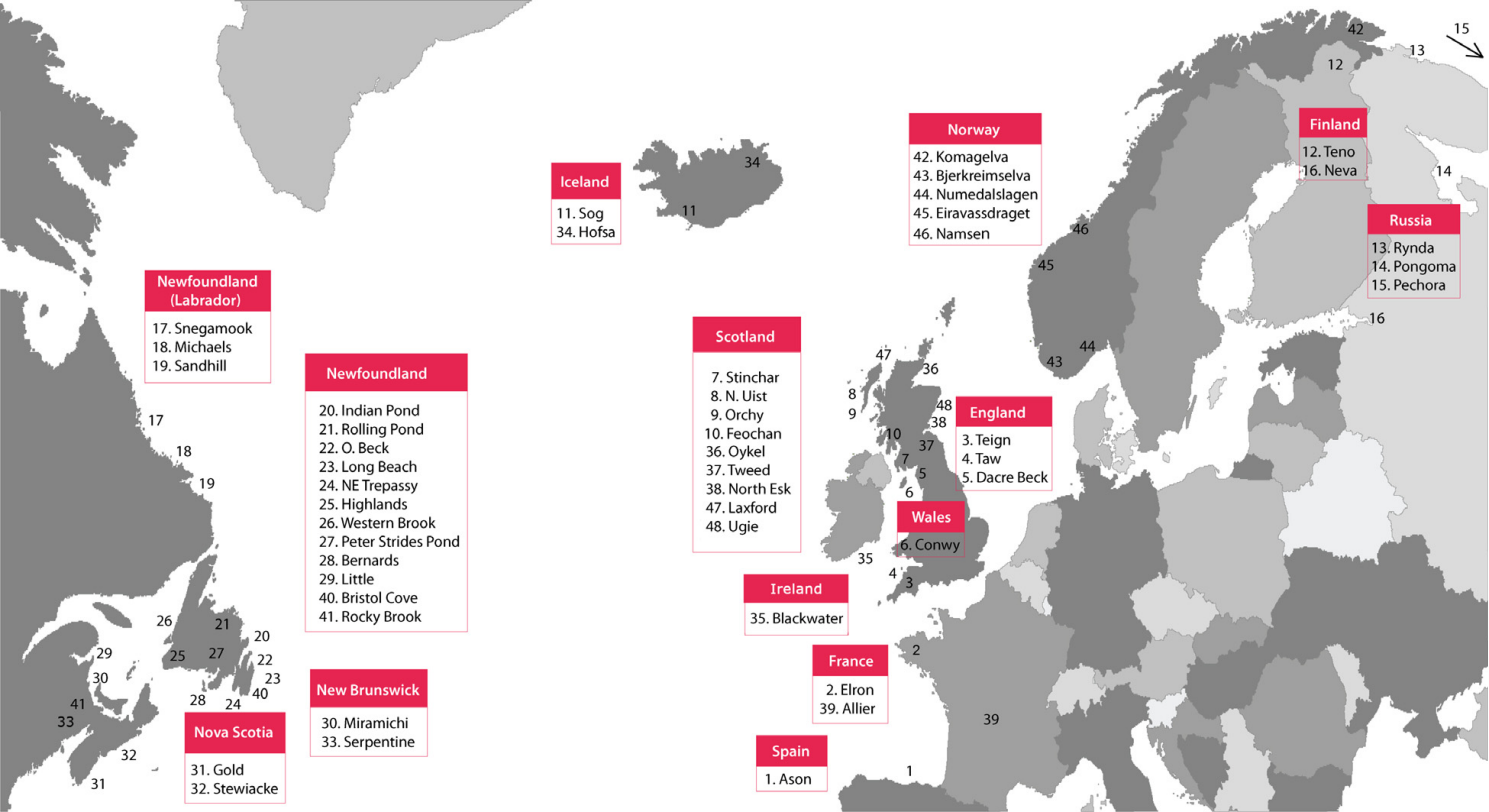
**A map showing the sampling locations**. See also Additional file 1, Table S1: Sampling sites, the number of samples and the division into groups.

**Figure 2:**
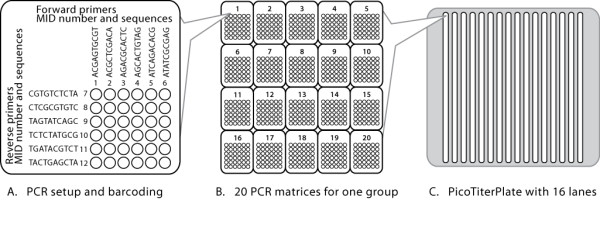
**The experimental setup**. In order to sequence 20 mitochondrial amplicons from 576 samples of Atlantic salmon, brown trout and Arctic charr, in a single run, the isolated DNA was divided into 16 groups, each containing 36 samples. The mitochondrial fragments were amplified from each group in a matrix of PCR reactions, using barcoded primers (MIDs) (A). 20 matrices of PCR reactions, one for each mitochondrion fragment, were carried out (B). Up to 720 amplicons from each group were pooled together in near equimolar concentrations. The 16 pools created were used to generate single stranded fragment DNA templates for the FLX sequencing. 16 amplified sst libraries, consisting of DNA fragments on beads, were loaded onto a PicoTiterPlate equipped with a sixteen-lane gasket where each library was assigned one lane (C). Based on the combination of MIDs on both sites of the amplicons within each group (library), individual sequencing reads were assigned to the corresponding samples.

546 *S. salar *samples derived from across the species' North Atlantic range were included in this study (Figure 1). In addition, 12 brown trout and 18 Arctic charr samples were included in the sequencing setup as a part of another study. The samples were divided into 16 groups, each composed of 36 samples (see Additional file 1, Table S1). Ten regions of the *S. salar *mitochondrial genome covering the D-loop region and nine coding genes (ND1, ND2, COXI, COXII, ATP6, ND3, ND4, ND5, CYTB) were selected for the SNP detection (Table 1), taking into account those regions previously shown to have high levels of polymorphism by studies of EST libraries and genome sequencing [19,20,22]. Some of the target regions were divided into 2-3 amplicons of about 400 bp, which is the average sequence length obtained with the FLX Titanium chemistry (Table 1). Thus, each sample was subjected to a total of 20 PCR reactions. For each of the 16 sample groups, 720 PCR reactions were carried out separately (Figure 2). Single PCR products of the expected sizes were obtained from the majority of the 11,520 reactions performed. Only 127 PCR reactions (1.1%) with *S. salar *DNA yielded low quantities of amplified products, despite optimization. Geographic bias was not pronounced; although the amplification of approximately half of the samples from two rivers produced low yields. This could be attributed to limited or low quality DNA isolated from the corresponding samples (data not shown).

**Table 1:** Target regions used in this study and the number of validated SNPs

Region	Region size including primer binding sites (bp)	Amplicon number	Amplicon size excluding primer binding sites (bp)	Number of SNPs	**SNPs per base**^**-1**^	Average number of reads supporting the SNPs per individual	Multiallelic SNPs	Transitions	Transversion	Indels
DLOOPB	423	1	381	21	0,055118	25.4 (±10.3)	1	11	8	2

ND1	1161	2	384	15	0,039063	28.0 (±16.3)	1	13	2	
		3	369	5	0,013550	28.2 (±12.3)		5		
		4	324	8	0,024691	13.9 (±5.8)		8		

ND2	770	5	361	13	0,036011	34.1 (±11.7)		13		
		6	346	7	0,020231	29.1 (±17.1)		5	2	

COXI	821	7	372	12	0,032258	21.2 (±11.6)		12		
		8	382	12	0,031414	40.9 (±14.2)		11	1	

COXII	715	9	361	7	0,019391	48.0 (±19.3)		7		
		10	311	7	0,022508	17.3 (±7.6)		7		

ATP6	414	11	375	13	0,034667	35.9 (±14.1)		11	2	

ND3	403	12	357	10	0,028011	30.5 (±12.8)		9	1	

ND4	1181	13	363	12	0,033058	27.6 (±11.6)		12		
		14	361	13	0,036011	26.7 (±12.5)		13		
		15	370	13	0,035135	19.3 (±9.8)		12	1	

ND5	783	16	345	8	0,023188	21.3 (±8.4)		8		
		17	370	12	0,032432	20.5 (±8.5)		12		

CYTB	1161	18	366	10	0,027322	35.9 (±14.6)		10		
		19	352	8	0,022727	14.5 (±6.2)		8		
		20	365	12	0,032877	25.6 (±9.9)	1	9	3	

Totals	7832		7215	218	0,030215	27.3 (±11.7)	3	196	20	2

The amplicon sequences without primers within each region do not overlap (see Table in Additional material 4). Accordingly, the total number of bp analyzed from the mitochondrion DNA was 7215.

### Pyrosequencing and Amplicon Analysis

The PCR products for each group were pooled for sequencing in near equimolar concentrations and the sequencing was carried out as described in Materials and Methods. The pyrosequencing yielded a total of 179,826,884 filter passed base pairs. An average of 11,546,081 bp (±1,612,536 SD) was obtained from each of the 16 regions on the picotiter plate, excluding one that yielded 6,635,668 bp. The majority of sequence reads were between 400-420 bp, corresponding to the size of the amplicons (see Additional file 2, Figure S1 and Table S2, on the distribution of read length). Each PCR product contained multiplex identifying sequences [MIDs], designed by Roche (454 Life Sciences), at both ends, which enabled the identification of the corresponding sample within a group (Figure 2). Hence, to correctly assign a PCR product to a sample, its entire sequence was required, including both MIDs. The GS Amplicon Variant Analyzer software from Roche, 454 Life Sciences was used to align and assign the sequence reads. The sequences obtained were assigned to 10,765 of the 10,920 *S. salar *products targeted by PCR (98.6%). The number of sequence reads (sequence coverage) supporting a SNP per individual was on average 27.3 with a standard deviation of 11.74. The yield of sequenced *S. salar *amplicons was generally high, with less than 1.5% of samples missing on average. The exception to this observation was amplicon 18 (Table 1), where no sequences were obtained for 62 of 546 *S. salar *samples (12%).

### SNP Analysis

**Figure 3:**
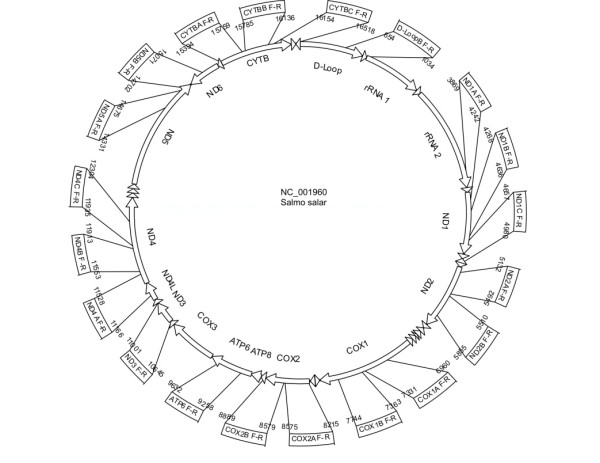
**A map of the *Salmo salar *mitochondrial genome**. The regions selected for sequence analysis in this study are shown.

**Figure 4:**
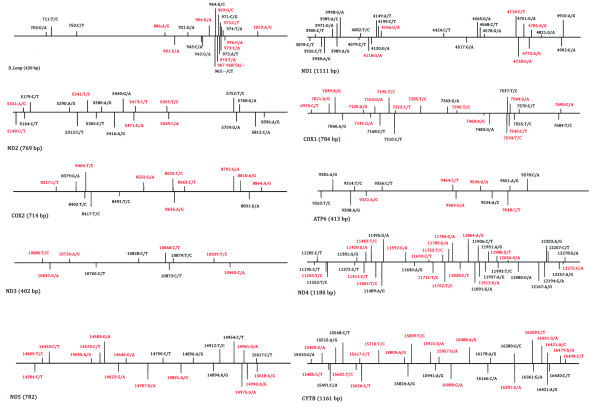
**Maps of the accepted SNPs obtained in this study, shown for each region**. New SNPs are shown red.

The GS Amplicon Variant Analyzer software identified a total of 1714 variants following alignment of the raw sequence data. Applying the first stringency filter, requiring sequence reads from both DNA strands, reduced this number to 904. After rejecting variants resulting from potential sequencing errors, including under-reads of homopolymer regions, and single base insertions, the variants were confined to those confirmed in at least 90% of replicate sequence reads. Subsequently, 242 variants remained. Thereof, 24 variants were rejected since they occurred in only one individual. Consequently, the alignment analysis and filtering yielded a total of 218 variants, consisting of 216 SNPs and 2 indels. Maps of the *S. salar *mitochondrial genome, indicating the regions selected for the SNP analysis and the accepted SNPs for each region are shown in Figure 3 and 4, respectively. (See also Table 1, and Additional file 3, Table S3, for further details on 454 read statistics). The greatest number of SNPs was observed in the ND4 gene region (38), whilst the lowest number (10) was observed in the ND3 gene. When considered as a proportion of the length of each region, the D-loop had the highest density of SNPs (0.55 SNPs bp^-1^) whilst the COX2 region gene yielded the least (0.021 SNPs bp^-1^). Of the variants observed, 196 are transitions while 20 are transversions, and nearly half of the latter is located in the D-Loop region (Table 1). In three cases (D-Loop: bp973; ND1: bp3989 and CYT-B: bp16421) both a transition and transversion were observed at the same locus in different samples. Indels were only observed within the D-Loop region, with a two bp insertion (CT) at bp 963 and a two bp deletion at bp 967. When compared to previous studies of smaller [20] and more geographically constrained discovery panels [15,19,22-24], 109 of the SNPs and one of the indels had been reported, while 107 new SNPs and one indel were identified in this study (Figure 4). When this data is considered according to populations, 0.5% of SNPs were found in all geographic locations, whilst 13.1% existed in only one. Additionally, a further 56.3% were found in less than 10 populations, whilst 13.1% were found in more than 50% of populations.

## Discussion

Next generation sequencing technology, such as 454 sequencing, is rapidly expanding the possibility for SNP mining by genome sequencing within a reasonable budget. However, the technology to-date has generally been restricted to the sequencing of large regions of the genome using relatively few samples [12,13]. In this work, a large SNP ascertainment panel was obtained at a relatively low cost by amplifying multiple regions of a mitochondrial genome in numerous samples and their subsequent sequencing using the 454 technology. The resultant SNP panel provides the least likelihood of ascertainment bias [25], with a discovery panel of 546 samples from 48 locations across the North Atlantic range of *S. salar*, making this the most informative and geographically comprehensive SNP panel for the species to-date (Figure 1).

The cost of sequencing using the 454 platform was minimized by carefully designing the experiment to require only a single pyrosequencing run, whilst obtaining sufficient sequence reads in both directions to yield a robust result, essentially minimizing false positives and avoid missing true polymorphisms from each ~400 bp read. The most challenging aspect faced was the need to ensure that each individual amplicon of a given type could be traced to the original sample. This was achieved using a sequencing plate with a 16-region gasket, and a set of 5' MID primer sequences. To decrease the primer cost (and ultimately the overall cost), the necessary adaptor sequences for the 454 sequencing were ligated to the PCR products instead of including them in the primer sequences; this may have also reduced complications in the PCR reactions due to the shorter 5' non-complementary primer sequences. Thus, sequencing libraries were prepared for the 16 PCR pools using the GS FLX Titanium General Library Preparation protocol for shotgun sequencing, instead of using amplicon sequencing (Roche, 454 Life Sciences). Furthermore, a Taq-comparable polymerase was used for generating the PCR pools of 11,520 reactions, instead of using an expensive high fidelity polymerase. The lack of proof-reading activity during the PCR might have increased the error rate in the sequence data, although the numerous specimens used here as well as the sequence coverage obtained should adequately compensate for this. Furthermore, variations observed in a single individual were not accepted as SNPs in accordance with the set criteria. Even if legitimate SNPs were necessarily discarded in this manner, their low frequency suggests that any information that was lost was probably minimal. Given that there is no geographical or population association, it can be assumed that this would not compromise the informative essence of the overall SNP suite, at least from the perspective of the studies of biodiversity and phylogeography. Considering the high coverage obtained in this experiment, the application of a high fidelity polymerase could enable the reliable SNP detection for simultaneous processing of even greater sample numbers than in the current study. Thus, if the budget is not restricting, the processing capacity of the method can be further increased by applying a high fidelity polymerase to generate the amplicons, at the cost of sequence coverage.

The sequence yield in this experiment was approximately 179 million bp, which is within the range of 160-320 million bp expected for Titanium 454 sequencing, using a 16-region gasket (Roche, 454 Life Sciences). However, for an undetermined reason, the sequence yield ranged from 6,635,668 to 13,135,520 filter-passed base pairs between different regions of the sequencing plate. Also, the sequence yield for amplicon 18 was relatively low in comparison to that of the other amplicons. This could be due to a bias in the sample pooling. Nevertheless, the distribution of sequences was relatively similar over the sample range, resulting in an average coverage of 27.3 (±11.7) sequence reads per SNP per individual. The sequencing setup described here also included 30 brown trout and Arctic charr samples. The data sets obtained for these samples were not included in this analysis, and they will be the subject of future studies. Nevertheless, they are mentioned here since they were a part of the original experimental setup of 576 individuals divided into 16 groups, each with 36 individuals. The total number of base pairs that were to be sequenced in this study (including the brown trout and Arctic charr samples) was 4,115,840 bp (number of sequenced bases for 576 individuals). The total sequence data obtained consisted of 179.826.884 bp, so 39-fold coverage would therefore be expected. However, a substantial part of the sequence reads were not assigned to any sample and were consequently excluded from the analysis. The anticipated reason behind this is accounted to short sequence reads missing a MID from either end. Fusing MID sequences to only one end of an amplicon should reduce this problem and increase the number of analysed sequence reads. This practice, however, reduces the number of samples, which can be analysed simultaneously, although with the emergence of new MID sequences validated for 454 sequencing, more samples with only one MID could be assigned.

Although the existence of MIDs on both ends of a sequence read was requisite in order to assign it to the corresponding individual, some global and consensi alignments (made with the GS Amplicon Variant Analyzer) included partial sequences. This was the result of default stringency levels causing parts of some sequences with low quality bases to be discarded. The trimming was however performed following the alignment of the sequence reads. Hence, as the design of the MIDs allows for (corrects) two sequencing errors, these partial sequences could still be assigned to the corresponding individual prior to the trimming.

As expected for high-throughput sequencing methods, which produce high amounts of reads, numerous deviations from the reference sequence were observed in the raw data. The stringent criteria applied here for the SNP validation rejected the great majority of these variants. However, the filtering may also have eliminated legitimate SNPs. Such variants could be identified by a modest reduction in the stringency levels and verified by Sanger sequencing. Following the validation, about 3.0% of the bases screened exhibited sequence variance and the final number of variants, 218 (including two indels), is nearly double the number of mtDNA SNPs previously reported. The new SNPs are predominantly transitions although the number of transversions is relatively high in the non-coding D-loop region (Table 1). Similar transition/transversion ratios were observed in other studies on the genetic variability of the *S. salar *mitochondrion [19,20,22]. The 107 new SNPs and a new indel identified in this study are indicated in Figure 4 in red letters. Although the average number of sequence reads supporting the new SNPs per individual is similar to that of those reported, the number of individuals harbouring the new SNPs is generally lower (see Additional file 3, Table S3). This is due to the numerous samples and the great geographical range analyzed here, which consequently allowed the detection of new rare variants. The greater the number of amplicons and individuals screened, the more likely it is that an accurate picture of the extent and nature of SNP variation will be revealed, and that a suite of SNP markers for population genetic studies can be compiled. The challenge remains to determine the optimal balance between the number of amplicons (i.e. proportion of mtDNA genome screened), the number of populations (i.e. representativeness of species as a whole) and the number of individuals per population for studies of matrilineal variation in the target species, Atlantic salmon. This will only be achieved by a detailed analysis of the within and among population variation and a consideration of the robustness of its evolutionary and phylogeographic implications. It should, however, provide a basis for robust inferences concerning the general levels of genetic diversity in the species and broad scale, deep phylogenetic structuring. Given, though, that the geographical sampling is extensive rather than intensive, and that sample sizes per population are limited, the ability to resolve any finer scale, shallower evolutionary structuring on the regional level will be diminished. To achieve the latter, a more focused and intensive search, based on sequencing of the entire mtDNA genome from more individuals would be of greater use since a higher proportion of regionally or population restricted or low frequency polymorphisms could be detected.

The analysis of within and among population distribution of variation is currently underway and can be expected to yield considerable insights into the phylogeography and matrilineal evolution of the Atlantic salmon. Analysis of the linkage of variation and its spatial distribution should also assist in the identification of a subset of the overall SNP suite that can be genetically typed more cost-effectively using PCR-based SNP assays. The SNP subset could then be investigated within samples from a wider geographical range and in archival material, in order to establish a more accurate assessment of the mixed stock assignment and population evolutionary hypotheses extracted from the current data set. Additionally, the markers that were identified in the study will likely prove highly useful as genetic tags for use in the assessment of the extent of population processes such as female mediated gene flow, as well as for tracking individual family groups in experimental studies.

## Conclusion

A novel approach that combines targeted PCR amplification and a single HT sequencing run proved successful in screening numerous targeted genomic regions in a large number of individuals. This technique presents a valuable tool for the identification of SNPs for population studies in other species, and also minimizes the risks of ascertainment bias associated with previous approaches that screened either confined regions of the genome or only several individuals from few populations.

## Methods

### Samples and DNA Extraction

Atlantic salmon samples (N = 546) were collected from 48 locations throughout the North Atlantic range of the species using electro-fishing. Additionally, 12 brown trout (*Salmo trutta*) and 18 Arctic charr (*Salvelinus alpinus*) samples were included in the sequencing run as a part of the experimental setup shown in Figure 2. Table S1 submitted as Additional file 1 lists the sampling sites, the number of samples, and their subdivision into 16 groups. DNA was isolated from fin-clip tissue samples using the DNeasy Blood & Tissue Kit (Qiagen) and standard protocols, and DNA concentrations were measured with a NanoDrop ND1000 spectrophotometer. The concentrations of stock DNA eluted were 9-484 ng/μl and diluted to 2-10 ng/μl for subsequent PCR reactions.

### Primer Design

Ten regions of the *S. salar *mitochondrial genome (DLOOPB, ND1, ND2, COXI, COXII, ATP6, ND3, ND4, ND5, CYTB) were selected for SNP detection (Table 1). Seven of the ten selected gene regions were amplified in two or three fragments of approximately 400 bps, resulting in a total of 20 amplicons covering 7,215 bps excluding the primer binding sites (Table 1). Primers were designed according to the reference sequence (16.7 kb) of the *S. salar *mitochondrion (NC_001960.1) (see Additional File 4, Table S4). Barcodes or multiplex identifying sequences (MIDs) of 10 bp were added to the 5' end of each primer, providing a unique means of identification for every sample in a single sequence group. The MIDs used (Figure 2), were those designed by Roche, 454 Life Sciences for automated software identification of samples following sequencing and allow up to 2 sequencing errors in the MID region before a read is misidentified. To minimize the number of MID tagged primers required, samples were divided into 16 groups, each composed of 36 individuals (see Additional File 1, Table S1, for subdivision of samples). Six forward MID-primers (numbered 1-6) and six reverse MID-primers (numbered 7-12) were synthesized (Sigma Aldrich) for each of the 20 amplicons. As a consequence of using a combination of 6 forward and 6 reverse MID tagged primers, the same MIDs could be re-used by conducting the PCR and sequencing of each of the 16 groups separately (See primer matrix, Figure 2). Critically, this minimized the cost associated with the use of multiple MID tagged primers.

### PCR Setup

In order to simultaneously amplify 20 traceable mitochondrial fragments from 576 samples in a single run, PCR reactions for each group of 36 samples were conducted separately, resulting in a total of 11,520 PCR reactions across all 16 groups. PCR was performed in 20 μl reactions containing 0.5 mM betaine (Sigma Aldrich), 50 μM dNTP mix, 1 × reaction buffer and 1 U Teg polymerase (Matís production, Taq comparable polymerase), 0.5 μM each primer (synthesized by Sigma Aldrich) and 2-3 μl DNA (2-10 ng/μl). Initial denaturation was at 94°C for 2 minutes followed by 31-33 cycles of 94°C for 60 seconds, 53°C for 45 seconds and 72°C for 90 seconds, and then a final extension of 5 minutes at 72°C. The relative amount of PCR products was estimated using gel electrophoresis and ethidium bromide staining. Subsequently, products from each group were pooled in near equimolar concentrations resulting in 16 pools of up to 720 amplicons.

### Construction of single stranded DNA libraries

The DNA pools (3 μg) were electrophoresed on 1% agarose gels and a band of approximately 400 bp was purified from each pool using the QIAquick Gel Extraction Kit (Qiagen). These were subsequently examined using Bioanalyzer 2100 and a DNA 7500 LabChip (Agilent Technologies). Observed traces were correlative for all 16 sample groups, with an average fragment length of 425 bp. Both ends of the DNA fragments were repaired, phosphorylated and ligated to adaptor oligonucleotides A and B. The DNA fragments carrying the 5'-biotin of adaptor B were immobilized onto magnetic streptavidin coated beads. Single stranded template DNAs carrying adaptor A at the 5'-end and adaptor B at the 3'-end were then purified by alkaline denaturation. Reagents, enzymes and oligonucleotides for the single-stranded DNA (sst DNA) library construction were supplied with the GS FLX Titanium General Library Preparation Kit (Roche, 454 Life Sciences). The integrity and concentrations of the sst libraries were estimated using the Bioanalyzer and the RNA 6000 LabChip and Quant-iT Ribogreen DNA Assay Kit (Invitrogen), respectively.

### 454 pyrosequencing and assembly

Shotgun sequencing of the 16 sst DNA libraries was carried out using the GS FLX Titanium reagents as described by the manufacturer (Roche, 454 Life Sciences). Purified DNA fragments were hybridised onto DNA capture beads and the 16 sst DNA libraries were separately amplified by emulsion PCR. Beads containing amplified DNA were deposited onto a 75 × 75 mm Titanium PicoTiterPlate equipped with a sixteen-region gasket. Those corresponding to each of the 16 original DNA pools (20 amplicons from 36 individuals) were assigned one region and then the pyrosequencing was performed in a single run. The sequence data generated from each region, corresponding to each of the 16 groups, was assembled separately using the GS Amplicon Variant Analyzer software (Roche, 454 Life Sciences) with default stringency settings. Sequence reads were sorted according to the sequence and the combination of the MIDs, and the sequence reads from an individual for each amplicon were aligned with the *S. salar *reference sequence (NC_001960.1).

### SNP analysis and mapping

Candidate SNPs were identified and checked against the global and consensus alignments of the corresponding sequence using the GS Amplicon Variant Analyzer software (Roche, 454 Life Sciences). Variants resulting from potential sequencing errors, including under-reads of homopolymer regions, and single base insertions (carry-forward events) were rejected [26]. Homopolymer under-reads and carry-forward events were observed in the misalignment of underlying consensi, by confirming support in both forward and reverse reads and, where necessary, the observation of underlying flowgrams. SNPs were accepted as valid only if they met the following criteria: (i) Sequence reads with variants were produced from both DNA strands. (ii) Those with >90% support from sequencing reads should have a total of more than 10 supporting reads in both read directions, or in cases of less than 10 supporting reads that the SNP in question should also be present in other samples with higher support. (iii) The final list of SNPs rejected those only found in one sample as these readings could be considered uninformative and possibly arose as a result of a PCR or sequencing error. To determine the sequence coverage for each SNP per individual, only sequence reads comprising the consensus sequence, truly supporting the SNP were taken into account, excluding those reads that did not show the variance (e.g. due to a sequencing error or truncation).

## Availability

The SNPs identified and evaluated in this study have been deposited in the National Center of Biotechnology (NCBI) SNP database (dbSNP) under submitter handle MATIS. The accession numbers are: NCBI_ss 295476608 - 295476815.

## Authors' contributions

The study was conceived and coordinated by SH and EV and set up by OF, ST and EV. DK, CGL and EV were responsible for the DNA collections. SM, SB, and GO organized and performed the PCR and the sequencing work. OF and SM prepared the sequence alignments and ST and KO did the SNP validations and variant analysis. OF, EV, SB, KO, ST, CGL and SC wrote the manuscript. All participants of this study read and approved the final draft.

## Acknowledgements

This work is part of the EU SALSEA MERGE project (No. 212529, FP7-ENV-2007-1) and was sponsored by EU funding in association with internal fundings from Matis and Marine Scotland. Their support is gratefully acknowledged. We thank Vidar Wennevik for providing samples from Norwegian populations for the work. Most of the remaining samples used were drawn largely from archival material collected over the last two decades for other purposes, by numerous individuals. Their contribution to making the study possible is also acknowledged.

## Supplementary Material

Additional file 1 ***Table S1: Sampling locations***. The file shows the sampling locations and the number of individuals per location as well as grouping of samples Click here for file

Additional file 2 ***Figure S1: Distribution of read length Table S2: Read length statistics of the FLX sequencing run***. The file contains a graph showing the number of reads vs. read length and a table indicating the read length statistics of the FLX sequencing run. Click here for file

Additional file 3 ***Table S3: mtDNA SNPs validated in this study***. The file shows the validated SNPs, their location according to *S. salar *mitochondrial DNA reference sequence (NC_001960.1); the number of individuals supporting the SNP; and the sequence coverage per individual. Click here for file

Additional file 4 ***Table S4: The primer set for the 20 amplicons used in this study***. The file shows the designation and the sequence of the primers used, as well as the primer binding sites on the mitochondrial DNA according to the reference sequence (NC_001960.1). Click here for file
