# Supplementary material for: Detection and mapping of mtDNA SNPs in Atlantic salmon using high throughput DNA sequencing
Source: BMC Genomics. 2011 Apr 7;12:179. doi: 10.1186/1471-2164-12-179 (PMC3079667; doi:10.1186/1471-2164-12-179)
Supplement: Additional file 1 — Table S1: Sampling locations. The file shows the sampling locations and the number of individuals per location as well as grouping of samples [file 1471-2164-12-179-S1.DOCX]

| **Additional File 1**  Table S1: Sampling sites, the number of samples and the division into groups | | | |  |  |
| --- | --- | --- | --- | --- | --- |
|  |  |  |  |  |  |
| River | Abbreviation | Location | Sample Size | Group | Sample number |
| Ason | AS | Spain | 12 | 1 | 1-36 |
| Elron | EL | France | 12 |  |  |
| Teign | TE | England | 6 |  |  |
| Taw | TA | England | 6 |  |  |
| Dacre beck | DB | England | 12 | 2 | 37-72 |
| Conwy | CW | Wales | 12 |  |  |
| Stinchar | ST | Scotland | 12 |  |  |
| N.Uist | NU | Scotland | 12 | 3 | 73-108 |
| Orchy | OR | Scotland | 12 |  |  |
| Feochan | F | Scotland | 12 |  |  |
| Sog | SG | Iceland (South) | 12 | 4 | 109-144 |
| Teno | TN | Finland | 12 |  |  |
| Rynda | RY | Russia | 12 |  |  |
| Pongoma | PG | Russia | 12 | 5 | 145-180 |
| Pechora | PE | Russia | 12 |  |  |
| Neva (Kymijoki) | NE | Finland (Baltic) | 12 |  |  |
| Snegamook | SN | Newfoundland (Labrador) | 12 | 6 | 181-216 |
| Michaels | MC | Newfoundland (Labrador) | 12 |  |  |
| Sandhill | SH | Newfoundland (Labrador) | 12 |  |  |
| Indian pond | IP | Newfoundland | 12 | 7 | 217-251 |
| Rolling pond | RP | Newfoundland | 12 |  |  |
| O. beck | OB | Newfoundland | 11 |  |  |
| Long beach | LB | Newfoundland | 8 | 7 8 | 252, 253-283 |
| NE Trepassy | NT | Newfoundland | 12 |  |  |
| Highlands | HG | Newfoundland | 12 |  |  |
| Western brook | WB | Newfoundland | 12 | 8 9 | 284-288 289-317 |
| Peter Strides pond | PS | Newfoundland | 12 |  |  |
| Bernards | BE | Newfoundland | 6 |  |  |
| Little | LT | Newfoundland | 6 |  |  |
| Miramichi | M | New Brunswick | 12 | 9 10 | 318-324 325-355 |
| Gold | G18 | Nova Scotia | 12 |  |  |
| Stewiacke | SW | Nova Scotia | 12 |  |  |
| Serpentine | SP | New Brunswick | 12 | 10 11 | 356-360 361-385 |
| L. Rannoch * | RC | Scotland | 12 |  |  |
| L.Earn * | EC | Scotland | 6 |  |  |
| L. Laidon * | LP | Scotland | 12 | 11 12 | 386-396 397-421 |
| Hofsa | HF | Iceland (Northern) | 12 |  |  |
| Blackwater | BV | Scotland | 12 |  |  |
| Oykel | OY | Scotland | 12 | 12 13 | 422-432 433-457 |
| Tweed | TW | Scotland | 12 |  |  |
| North Esk | G9 | Scotland | 12 |  |  |
| Allier | AG | France | 12 | 13 14 | 458-468 469-504 |
| Bristol Cove | BC | Newfoundland | 12 |  |  |
| Rocky brook | RB | Newfoundland | 12 |  |  |
| Komagelva | KO | Norway | 11 |  |  |
| Bjerkreimselva | BJ | Norway | 12 | 15 | 505-539 |
| Numedalslagen | NU | Norway | 12 |  |  |
| Eiravassdraget | ER | Norway | 12 |  |  |
| Namsen | NA | Norway | 12 | 16 | 540-576 |
| Laxford | LX | Scotland | 12 |  |  |
| Ugie | UF | Scotland | 12 |  |  |
|  |  |  | 576 |  |  |
| **Brown Trout and Arctic Charr* | |  |  |  |  |
