# Supplementary material for: Detection and mapping of mtDNA SNPs in Atlantic salmon using high throughput DNA sequencing
Source: BMC Genomics. 2011 Apr 7;12:179. doi: 10.1186/1471-2164-12-179 (PMC3079667; doi:10.1186/1471-2164-12-179)
Supplement: Additional file 2 — Figure S1: Distribution of read length Table S2: Read length statistics of the FLX sequencing run. The file contains a graph showing the number of reads vs. read length and a table indicating the read length statistics of the FLX sequencing run. [file 1471-2164-12-179-S2.DOCX]

**Additional File 2**

Figure S1: Distribution of read length – Number of reads vs. read length


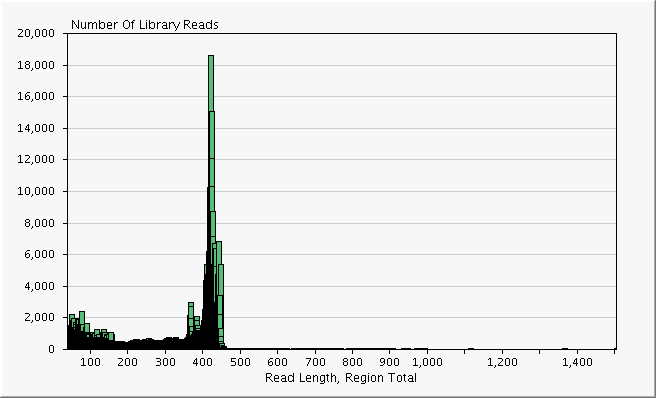


Table S2: Read length of filter-passed sequences following

the 454 sequencing run - Statistics*

| Length statistics - Combined data  of 16 sequencing Lanes | Length in bp |
| --- | --- |
| Length Average | 320.74 |
| Longest Reads Length | 1504 |
| Shortest Reads Length | 40 |
| Median Reads Length | 402.0 |

*Only a part of the filter-passed sequence reads, i.e. sequences of sufficient read length containing MID sequences at both ends, were processed with the GS Amplicon Variant Analyzer software.
