# Supplementary material for: Detection and mapping of mtDNA SNPs in Atlantic salmon using high throughput DNA sequencing
Source: BMC Genomics. 2011 Apr 7;12:179. doi: 10.1186/1471-2164-12-179 (PMC3079667; doi:10.1186/1471-2164-12-179)
Supplement: Additional file 3 — Table S3: mtDNA SNPs validated in this study. The file shows the validated SNPs, their location according to S. salar mitochondrial DNA reference sequence (NC_001960.1); the number of individuals supporting the SNP; and the sequence coverage per individual. [file 1471-2164-12-179-S3.DOCX]

| **Additional File 3**  Table S3: List of identified and validated SNPs, mtDNA region, number of individuals and sequence depth | | | | | |  |  |  |  |
| --- | --- | --- | --- | --- | --- | --- | --- | --- | --- |
| **Region** | **Amplicon** | **SNP** | **Number of individuals supporting the SNP** | **Average number of reads supporting the SNPs per individual** | **Number of reads standard deviation** |  |  |  |  |
|  |  | 703:G/A | 55 | 20.36 | 11.94 |  |  |  |  |
|  |  | 711:T/C | 141 | 21.04 | 7.75 |  |  |  |  |
|  |  | 750:C/T | 39 | 25.10 | 12.34 |  |  |  |  |
|  |  | 886:A/G | 2 | 27.00 | 19.80 |  |  |  |  |
|  |  | 901:G/A | 4 | 25.00 | 12.25 |  |  |  |  |
|  |  | 921:G/A | 15 | 19.07 | 10.81 |  |  |  |  |
|  |  | 942:G/A | 536 | 24.20 | 10.00 |  |  |  |  |
|  |  | 943:C/A | 412 | 22.56 | 10.08 |  |  |  |  |
|  |  | 954:G/A | 35 | 28.51 | 12.15 |  |  |  |  |
| DLOOPB | 1 | 963.5:--/CT | 124 | 24.86 | 8.11 |  |  |  |  |
|  |  | 964:A/C | 46 | 28.17 | 11.92 |  |  |  |  |
|  |  | 967-968:TA/-- | 5 | 32.40 | 9.91 |  |  |  |  |
|  |  | 969:G/C | 140 | 24.54 | 8.10 |  |  |  |  |
|  |  | 970:T/A | 140 | 24.65 | 8.12 |  |  |  |  |
|  |  | 971:C/G | 140 | 24.58 | 8.10 |  |  |  |  |
|  |  | 972:A/T | 140 | 24.58 | 8.10 |  |  |  |  |
|  |  | 973:C/A | 3 | 21.00 | 11.27 |  |  |  |  |
|  |  | 973:C/T | 37 | 29.08 | 12.46 |  |  |  |  |
|  |  | 974:T/A | 140 | 25.05 | 8.24 |  |  |  |  |
|  |  | 976:G/A | 142 | 24.90 | 8.27 |  |  |  |  |
|  |  | 1029:A/G | 3 | 36.67 | 5.69 |  |  |  |  |
|  |  | 3878:C/T | 22 | 38.41 | 23.51 |  |  |  |  |
|  |  | 3900:G/A | 13 | 20.23 | 19.54 |  |  |  |  |
|  |  | 3926:C/T | 8 | 23.25 | 13.83 |  |  |  |  |
|  |  | 3938:A/G | 5 | 19.40 | 4.83 |  |  |  |  |
|  |  | 3971:G/A | 169 | 24.15 | 15.53 |  |  |  |  |
|  |  | 3989:A/G | 396 | 24.26 | 14.91 |  |  |  |  |
|  |  | 3989:A/C | 5 | 30.40 | 15.00 |  |  |  |  |
|  | 2 | 3998:G/A | 35 | 34.34 | 24.61 |  |  |  |  |
|  |  | 4079:C/T | 141 | 24.38 | 11.97 |  |  |  |  |
|  |  | 4082:T/C | 142 | 24.53 | 11.90 |  |  |  |  |
|  |  | 4118:G/A | 35 | 32.31 | 24.55 |  |  |  |  |
|  |  | 4130:G/A | 34 | 28.79 | 20.10 |  |  |  |  |
|  |  | 4149:A/T | 14 | 25.50 | 8.45 |  |  |  |  |
| ND1 |  | 4166:G/A | 21 | 40.62 | 24.40 |  |  |  |  |
|  |  | 4199:C/T | 6 | 35.50 | 12.01 |  |  |  |  |
|  |  | 4424:C/T | 136 | 29.66 | 12.58 |  |  |  |  |
|  |  | 4517:G/A | 121 | 29.16 | 14.49 |  |  |  |  |
|  | 3 | 4565:G/A | 149 | 25.73 | 10.49 |  |  |  |  |
|  |  | 4568:C/T | 145 | 29.92 | 12.49 |  |  |  |  |
|  |  | 4578:G/A | 10 | 26.70 | 11.59 |  |  |  |  |
|  |  | 4718:C/T | 5 | 13.00 | 2.74 |  |  |  |  |
|  |  | 4728:G/A | 3 | 14.67 | 7.02 |  |  |  |  |
|  |  | 4751:G/A | 154 | 14.45 | 6.23 |  |  |  |  |
|  | 4 | 4772:A/G | 7 | 15.43 | 8.00 |  |  |  |  |
|  |  | 4781:A/G | 45 | 15.22 | 5.95 |  |  |  |  |
|  |  | 4821:G/A | 331 | 12.91 | 6.52 |  |  |  |  |
|  |  | 4902:G/A | 4 | 13.00 | 3.56 |  |  |  |  |
|  |  | 4910:A/G | 46 | 12.74 | 6.04 |  |  |  |  |
|  |  | 5140:C/T | 3 | 44.33 | 19.76 |  |  |  |  |
|  |  | 5161:A/G | 3 | 47.00 | 2.00 |  |  |  |  |
|  |  | 5164:C/T | 111 | 37.15 | 15.37 |  |  |  |  |
|  |  | 5179:C/T | 5 | 32.40 | 8.47 |  |  |  |  |
|  |  | 5290:A/G | 10 | 32.50 | 9.83 |  |  |  |  |
|  | 5 | 5312:C/T | 8 | 30.87 | 10.13 |  |  |  |  |
|  |  | 5341:T/C | 14 | 25.14 | 12.89 |  |  |  |  |
|  |  | 5365:C/T | 109 | 36.43 | 16.70 |  |  |  |  |
|  |  | 5380:A/G | 8 | 30.38 | 10.28 |  |  |  |  |
|  |  | 5416:A/G | 157 | 38.27 | 16.86 |  |  |  |  |
| ND2 |  | 5440:G/A | 12 | 18.75 | 8.83 |  |  |  |  |
|  |  | 5471:G/A | 3 | 43.00 | 10.00 |  |  |  |  |
|  |  | 5473:C/T | 3 | 27.33 | 10.50 |  |  |  |  |
|  |  | 5563:T/C | 31 | 28.03 | 11.61 |  |  |  |  |
|  |  | 5569:C/A | 2 | 46.50 | 47.38 |  |  |  |  |
|  |  | 5752:T/C | 31 | 27.23 | 12.89 |  |  |  |  |
|  | 6 | 5759:G/A | 143 | 26.24 | 12.30 |  |  |  |  |
|  |  | 5768:G/A | 145 | 26.41 | 12.29 |  |  |  |  |
|  |  | 5812:C/A | 143 | 24.33 | 11.23 |  |  |  |  |
|  |  | 5836:A/G | 31 | 24.94 | 12.32 |  |  |  |  |
|  |  | 6970:C/T | 396 | 22.80 | 11.44 |  |  |  |  |
|  |  | 7021:A/G | 25 | 15.04 | 13.16 |  |  |  |  |
|  |  | 7039:A/G | 32 | 10.63 | 10.03 |  |  |  |  |
|  |  | 7060:A/G | 398 | 25.14 | 11.75 |  |  |  |  |
|  |  | 7120:A/G | 2 | 35.00 | 16.97 |  |  |  |  |
|  | 7 | 7141:G/A | 3 | 24.67 | 10.69 |  |  |  |  |
|  |  | 7153:G/A | 399 | 26.28 | 12.04 |  |  |  |  |
|  |  | 7168:C/T | 5 | 24.40 | 13.37 |  |  |  |  |
|  |  | 7195:T/C | 15 | 8.33 | 8.05 |  |  |  |  |
|  |  | 7210:C/T | 427 | 25.56 | 12.74 |  |  |  |  |
|  |  | 7213:C/T | 32 | 8.97 | 7.21 |  |  |  |  |
|  |  | 7285:T/C | 399 | 27.35 | 12.14 |  |  |  |  |
|  |  | 7363:G/A | 34 | 29.76 | 16.28 |  |  |  |  |
|  |  | 7390:T/C | 541 | 38.06 | 15.49 |  |  |  |  |
| COXI |  | 7468:A/G | 4 | 53.25 | 13.52 |  |  |  |  |
|  |  | 7483:G/A | 189 | 40.46 | 15.85 |  |  |  |  |
|  |  | 7534:T/C | 39 | 42.08 | 20.95 |  |  |  |  |
|  | 8 | 7537:G/A | 32 | 41.84 | 15.56 |  |  |  |  |
|  |  | 7540:C/T | 5 | 32.00 | 7.28 |  |  |  |  |
|  |  | 7544:G/A | 2 | 35.00 | 9.90 |  |  |  |  |
|  |  | 7555:T/C | 157 | 43.32 | 16.39 |  |  |  |  |
|  |  | 7570:C/T | 161 | 40.70 | 15.42 |  |  |  |  |
|  |  | 7684:T/C | 157 | 42.50 | 16.45 |  |  |  |  |
|  |  | 7690:C/A | 2 | 51.50 | 7.78 |  |  |  |  |
|  |  | 8327:C/T | 2 | 65.50 | 43.13 |  |  |  |  |
|  |  | 8379:G/A | 158 | 43.72 | 17.43 |  |  |  |  |
|  |  | 8402:T/C | 144 | 43.75 | 15.30 |  |  |  |  |
|  | 9 | 8404:T/C | 2 | 45.50 | 6.36 |  |  |  |  |
|  |  | 8417:T/C | 41 | 63.90 | 25.32 |  |  |  |  |
|  |  | 8492:T/C | 177 | 48.07 | 19.83 |  |  |  |  |
|  |  | 8556:G/A | 2 | 25.50 | 7.78 |  |  |  |  |
|  |  | 8633:T/C | 130 | 16.28 | 8.92 |  |  |  |  |
|  |  | 8636:A/G | 3 | 17.00 | 4.00 |  |  |  |  |
|  |  | 8663:C/T | 6 | 16.33 | 10.69 |  |  |  |  |
| COXII | 10 | 8792:G/A | 62 | 26.15 | 11.14 |  |  |  |  |
|  |  | 8810:A/G | 5 | 12.20 | 5.36 |  |  |  |  |
|  |  | 8831:G/A | 108 | 15.28 | 7.64 |  |  |  |  |
|  |  | 8864:A/G | 4 | 17.75 | 5.50 |  |  |  |  |
|  |  | 9263:T/C | 430 | 35.46 | 15.03 |  |  |  |  |
|  |  | 9281:A/G | 23 | 38.48 | 11.42 |  |  |  |  |
|  |  | 9308:A/G | 22 | 35.27 | 12.70 |  |  |  |  |
|  |  | 9314:T/C | 4 | 36.50 | 5.92 |  |  |  |  |
|  |  | 9332:A/G | 7 | 40.57 | 12.69 |  |  |  |  |
|  |  | 9356:C/T | 409 | 35.57 | 15.09 |  |  |  |  |
| ATP6 | 11 | 9464:C/T | 3 | 29.00 | 9.00 |  |  |  |  |
|  |  | 9469:G/A | 5 | 38.00 | 14.92 |  |  |  |  |
|  |  | 9506:G/A | 11 | 36.82 | 22.60 |  |  |  |  |
|  |  | 9524:A/C | 19 | 37.68 | 12.39 |  |  |  |  |
|  |  | 9548:C/T | 2 | 24.50 | 19.09 |  |  |  |  |
|  |  | 9551:A/G | 400 | 38.55 | 15.47 |  |  |  |  |
|  |  | 9578:C/A | 31 | 40.23 | 16.56 |  |  |  |  |
|  |  | 10686:T/C | 2 | 37.00 | 9.90 |  |  |  |  |
|  |  | 10697:G/A | 6 | 28.83 | 24.43 |  |  |  |  |
|  |  | 10726:A/G | 3 | 31.67 | 6.51 |  |  |  |  |
|  |  | 10765:C/T | 143 | 27.78 | 14.12 |  |  |  |  |
| ND3 | 12 | 10828:C/T | 395 | 30.36 | 14.79 |  |  |  |  |
|  |  | 10868:C/T | 5 | 35.40 | 10.71 |  |  |  |  |
|  |  | 10873:C/T | 160 | 26.54 | 14.01 |  |  |  |  |
|  |  | 10879:T/C | 113 | 29.18 | 14.90 |  |  |  |  |
|  |  | 10939:T/C | 22 | 19.36 | 9.89 |  |  |  |  |
|  |  | 10963:C/A | 3 | 38.67 | 9.02 |  |  |  |  |
|  |  | 11196:C/T | 405 | 31.71 | 15.24 |  |  |  |  |
|  |  | 11205:C/T | 5 | 29.00 | 4.69 |  |  |  |  |
|  |  | 11250:T/C | 8 | 13.88 | 13.32 |  |  |  |  |
|  |  | 11253:A/G | 397 | 32.84 | 15.17 |  |  |  |  |
|  |  | 11372:C/T | 8 | 30.63 | 8.00 |  |  |  |  |
|  | 13 | 11381:A/G | 7 | 33.86 | 5.24 |  |  |  |  |
|  |  | 11411:C/T | 9 | 13.11 | 13.14 |  |  |  |  |
|  |  | 11420:G/A | 539 | 31.98 | 14.42 |  |  |  |  |
|  |  | 11443:T/C | 4 | 42.50 | 15.76 |  |  |  |  |
|  |  | 11462:T/C | 12 | 12.17 | 7.80 |  |  |  |  |
|  |  | 11489:A/G  11495:G/A | 394  29 | 32.33  28.17 | 14.78  15.51 |  |  |  |  |
|  |  |  |  |  |  |  |  |  |  |
|  |  | 11592:G/A | 12 | 14.08 | 10.84 |  |  |  |  |
|  |  | 11630:A/G | 8 | 32.38 | 13.19 |  |  |  |  |
|  |  | 11693:C/T | 5 | 36.60 | 7.20 |  |  |  |  |
|  |  | 11711:T/C | 13 | 17.62 | 14.33 |  |  |  |  |
|  |  | 11753:T/C | 122 | 28.26 | 12.93 |  |  |  |  |
| ND4 | 14 | 11762:T/C | 15 | 29.67 | 15.17 |  |  |  |  |
|  |  | 11780:G/A | 13 | 20.00 | 16.70 |  |  |  |  |
|  |  | 11786:G/A | 14 | 19.64 | 14.69 |  |  |  |  |
|  |  | 11828:C/T | 12 | 15.75 | 11.98 |  |  |  |  |
|  |  | 11864:A/G | 2 | 35.00 | 2.83 |  |  |  |  |
|  |  | 11891:G/A | 156 | 29.46 | 13.62 |  |  |  |  |
|  |  | 11906:C/T | 144 | 30.42 | 13.07 |  |  |  |  |
|  |  | 11912:G/A | 3 | 36.33 | 12.74 |  |  |  |  |
|  |  | 11937:A/G | 404 | 21.38 | 10.43 |  |  |  |  |
|  |  | 11951:A/G | 141 | 19.87 | 9.30 |  |  |  |  |
|  |  | 11988:G/T | 545 | 22.80 | 11.44 |  |  |  |  |
|  |  | 11993:T/C | 157 | 21.39 | 11.12 |  |  |  |  |
|  |  | 12056:G/A | 2 | 13.50 | 9.19 |  |  |  |  |
|  | 15 | 12080:G/A | 142 | 19.50 | 9.48 |  |  |  |  |
|  |  | 12167:A/G | 145 | 17.59 | 8.73 |  |  |  |  |
|  |  | 12194:G/A | 157 | 19.24 | 10.73 |  |  |  |  |
|  |  | 12203:A/G | 141 | 17.77 | 9.11 |  |  |  |  |
|  |  | 12207:C/T | 141 | 17.07 | 8.90 |  |  |  |  |
|  |  | 12257:A/G | 4 | 16.00 | 6.63 |  |  |  |  |
|  |  | 12275:G/A | 24 | 27.08 | 12.20 |  |  |  |  |
|  |  | 12278:G/A | 157 | 18.14 | 10.03 |  |  |  |  |
|  |  | 14384:C/T | 2 | 13.00 | 5.66 |  |  |  |  |
|  |  | 14405:T/C | 103 | 19.06 | 7.94 |  |  |  |  |
|  |  | 14425:C/T | 4 | 35.50 | 5.97 |  |  |  |  |
|  | 16 | 14545:A/G | 3 | 19.00 | 11.36 |  |  |  |  |
|  |  | 14570:C/T | 141 | 22.13 | 10.49 |  |  |  |  |
|  |  | 14583:G/A | 5 | 15.00 | 7.31 |  |  |  |  |
|  |  | 14623:G/A | 3 | 25.33 | 8.50 |  |  |  |  |
|  |  | 14645:G/A | 144 | 21.46 | 10.21 |  |  |  |  |
|  |  | 14707:G/A | 8 | 32.25 | 12.75 |  |  |  |  |
| ND5 |  | 14750:C/T | 106 | 19.99 | 8.95 |  |  |  |  |
|  |  | 14801:A/G | 3 | 24.33 | 9.24 |  |  |  |  |
|  |  | 14836:A/G | 5 | 26.00 | 4.47 |  |  |  |  |
|  |  | 14894:A/G | 26 | 25.31 | 10.36 |  |  |  |  |
|  | 17 | 14912:T/C | 19 | 14.00 | 9.07 |  |  |  |  |
|  |  | 14954:C/T | 145 | 22.83 | 10.07 |  |  |  |  |
|  |  | 14975:G/A | 17 | 12.35 | 9.32 |  |  |  |  |
|  |  | 14981:G/A | 19 | 12.53 | 9.00 |  |  |  |  |
|  |  | 14990:A/G | 2 | 13.50 | 4.95 |  |  |  |  |
|  |  | 15017:C/T | 144 | 23.44 | 10.04 |  |  |  |  |
|  |  | 15018:A/G | 3 | 20.00 | 3.61 |  |  |  |  |
|  |  | 15410:G/A | 24 | 29.62 | 14.00 |  |  |  |  |
|  |  | 15450:G/A | 2 | 41.50 | 14.85 |  |  |  |  |
|  |  | 15485:C/T | 23 | 27.00 | 12.98 |  |  |  |  |
|  |  | 15491:C/A | 129 | 37.33 | 15.30 |  |  |  |  |
|  | 18 | 15515:A/G | 2 | 52.00 | 15.55 |  |  |  |  |
|  |  | 15548:C/T | 102 | 37.44 | 15.96 |  |  |  |  |
|  |  | 15602:T/C | 6 | 37.50 | 21.32 |  |  |  |  |
|  |  | 15617:C/T | 9 | 37.78 | 14.00 |  |  |  |  |
|  |  | 15626:C/T | 31 | 29.52 | 14.40 |  |  |  |  |
|  |  | 15710:T/C | 3 | 29.33 | 7.64 |  |  |  |  |
|  |  | 15809:A/G | 4 | 18.00 | 13.19 |  |  |  |  |
|  |  | 15824:A/G | 7 | 12.29 | 6.13 |  |  |  |  |
|  |  | 15839:T/C | 4 | 10.25 | 4.57 |  |  |  |  |
| CYTB | 19 | 15921:G/A | 8 | 14.25 | 6.45 |  |  |  |  |
|  |  | 15941:A/G | 103 | 13.49 | 6.42 |  |  |  |  |
|  |  | 15957:G/A | 2 | 20.50 | 3.54 |  |  |  |  |
|  |  | 16008:G/A | 14 | 13.43 | 4.75 |  |  |  |  |
|  |  | 16088:A/G | 2 | 5.50 | 0.71 |  |  |  |  |
|  |  | 16166:C/A  16178:A/G | 139  149 | 22.92  19.26 | 10.15  11.22 |  |  |  |  |
|  |  |  |  |  |  |  |  |  |  |
|  |  | 16280:G/C | 151 | 27.44 | 13.06 |  |  |  |  |
|  |  | 16281:G/A | 6 | 33.83 | 9.50 |  |  |  |  |
|  |  | 16358:C/T | 2 | 25.00 | 8.48 |  |  |  |  |
|  | 20 | 16361:G/A | 172 | 27.41 | 12.95 |  |  |  |  |
|  |  | 16401:G/A | 4 | 24.25 | 8.10 |  |  |  |  |
|  |  | 16421:A/C | 30 | 30.43 | 13.13 |  |  |  |  |
|  |  | 16421:A/G | 112 | 28.99 | 12.66 |  |  |  |  |
|  |  | 16430:C/T | 124 | 27.20 | 13.40 |  |  |  |  |
|  |  | 16479:G/A | 3 | 18.33 | 3.21 |  |  |  |  |
|  |  | 16498:C/T | 3 | 19.33 | 3.21 |  |  |  |  |
