# Supplementary material for: Detection and mapping of mtDNA SNPs in Atlantic salmon using high throughput DNA sequencing
Source: BMC Genomics. 2011 Apr 7;12:179. doi: 10.1186/1471-2164-12-179 (PMC3079667; doi:10.1186/1471-2164-12-179)
Supplement: Additional file 4 — Table S4: The primer set for the 20 amplicons used in this study. The file shows the designation and the sequence of the primers used, as well as the primer binding sites on the mitochondrial DNA according to the reference sequence (NC_001960.1). [file 1471-2164-12-179-S4.DOCX]

| **Additional File 4**  Table S4: The primers for the 20 amplicons analyzed in this study | | | |
| --- | --- | --- | --- |
| **Primer designation** | **Primer Sequence 5-3'** | **5' base position of primer target site** | **Length of primer target site** |
| DLOOPB-F | GAACCCATGTATCATGGTGG | 637 | 20 |
| DLOOPB-R | GGGTCCATCTTAACAGCTTCAG | 1059 | 22 |
| ND1A-F | CCTTAGCTATGACTACCACCC | 3838 | 21 |
| ND1A-R | GAGCTCCAATTAGGGCAT | 4260 | 18 |
| ND1B-F | CTAATTGGAGCTCTACGAGC | 4248 | 20 |
| ND1B-R | GCGGCCTTTGTTATTAGG | 4654 | 18 |
| ND1C-F | AACCTAATAACAAAGGCCGCCC | 4635 | 22 |
| ND1C-R | TGGGCCCGAAAGCTTAAT | 4998 | 18 |
| ND2A-F | CTGACTCCTTGCATGAATAGGC | 5110 | 22 |
| ND2A-R | GGCCTATCGCAATGAGTA | 5510 | 18 |
| ND2B-F | CTCTACTCATTGCGATAGGC | 5490 | 20 |
| ND2B-R | CTAGTGTGGCAGATAGTGGGAGTC | 5879 | 24 |
| COXIA-F | CTTCAATTCTTGGGGCCA | 6942 | 18 |
| COXIA-R | CATATGGTGGGCTCAAACGA | 7351 | 20 |
| COXIB-F | GCCCACCATATGTTTACTGTCGG | 7340 | 23 |
| COXIB-R | GTGCTGGGGAAAAAAGGT | 7762 | 18 |
| COXIIA-F | GCACATCCCTCACAACTAGGAT | 8193 | 22 |
| COXIIA-R | AAGACGAAATTGACCGGGC | 8594 | 19 |
| COXIIB-F | CACCCAAGACTTAACGCC | 8561 | 18 |
| COXIIB-R | CGCTATTCCCGATTTAGC | 8907 | 18 |
| ATP6-F | GCCTTATCACTCTCCAAGGA | 9238 | 20 |
| ATP6-R | AGGCTGCTGTAGCGATTAG | 9651 | 19 |
| ND3-F | CGGTCTTGGTTAGAATCCAAGG | 10623 | 22 |
| ND3-R | GGGTCTTGTTTTGGACTAACTGCC | 11025 | 24 |
| ND4A-F | CTTCTCTCAGCCCTTCTATG | 11146 | 20 |
| ND4A-R | TAGGGGGTCAGTTGCTAA | 11546 | 18 |
| ND4B-F | AACTGACCCCCTATCGACA | 11534 | 19 |
| ND4B-R | CCTCACGTTAAGAGGTGTATGG | 11935 | 22 |
| ND4C-F | GCCCATACACCTCTTAACGTGAG | 11912 | 23 |
| ND4C-R | GAGACCGTGTGCAATCATTAGG | 12326 | 22 |
| ND5A-F | CAACCCCTCCGTTATTAATCCC | 14309 | 22 |
| ND5A-R | GTTGTGACCATAGGTAGGTGAGTGGA | 14701 | 26 |
| ND5B-F | CTCACCTACCTATGGTCACAAC | 14680 | 22 |
| ND5B-R | GCTGCCGAACCTTTTCCAGA | 15091 | 20 |
| CYTBA-F | GAACCTTAATGGCCAACC | 15376 | 18 |
| CYTBA-R | AACGTAGCCTACGAAGGCAG | 15779 | 20 |
| CYTBB-F | TTCGTAGGCTACGTTCTTCC | 15765 | 20 |
| CYTBB-R | GGCAGGTGTAAAATTGTCTGGGTC | 16160 | 24 |
| CYTBC-F | CGGGGACCCAGACAATTTTAC | 16133 | 21 |
| CYTBC-R | GAGCTACTAGGGCAGGTTC | 16537 | 19 |

The file shows the designation and the sequence of the primers used, as well as the primer binding sites on the mitochondrial DNA according to the reference sequence (NC_001960.1). The 5’ MID sequences are not shown.
